# Health education and the control of intestinal worm infections in China: a new vision

**DOI:** 10.1186/1756-3305-7-344

**Published:** 2014-07-24

**Authors:** Donald P McManus, Franziska A Bieri, Yue-Sheng Li, Gail M Williams, Li-Ping Yuan, Yang Henglin, Zun-Wei Du, Archie CA Clements, Peter Steinmann, Giovanna Raso, Peiling Yap, Ricardo J Soares Magalhães, Donald Stewart, Allen G Ross, Kate Halton, Xiao-Nong Zhou, Remigio M Olveda, Veronica Tallo, Darren J Gray

**Affiliations:** QIMR Berghofer Medical Research Institute, Herston, Brisbane, Queensland Australia; The School of Population Health, The University of Queensland, Brisbane, Queensland Australia; Research School of Population Health, The Australian National University, Canberra, Australia; Hunan Institute of Parasitic Diseases, WHO Collaborating Centre for Research and Control on Schistosomiasis in Lake Region, Yueyang, China; Yunnan Institute of Parasitic Diseases, Pu’er, China; Swiss Tropical and Public Health Institute, University of Basel, Basel, Switzerland; School of Veterinary Science, University of Queensland, Brisbane, Gatton Australia; Griffith Health Institute, Griffith University, Brisbane, Australia; School of Public Health and Social Work, Queensland University of Technology, Brisbane, Australia; National Institute of Parasitic Disease, Chinese Center for Disease Control and Prevention, Shanghai, China; Research Institute for Tropical Medicine, Manila, Philippines

**Keywords:** *Ascaris lumbricoides*, *Trichuris trichiura*, *Necator americanus*, *Ancylostoma duodenale*, Soil-transmitted helminths (STHs), People’s Republic of China, Health education, “Magic Glasses” video

## Abstract

**Background:**

The transmission of soil-transmitted helminths (STHs) is associated with poverty, poor hygiene behaviour, lack of clean water and inadequate waste disposal and sanitation. Periodic administration of benzimidazole drugs is the mainstay for global STH control but it does not prevent re-infection, and is unlikely to interrupt transmission as a stand-alone intervention.

**Findings:**

We reported recently on the development and successful testing in Hunan province, PR China, of a health education package to prevent STH infections in Han Chinese primary school students. We have recently commenced a new trial of the package in the ethnically diverse Xishuangbanna autonomous prefecture in Yunnan province and the approach is also being tested in West Africa, with further expansion into the Philippines in 2015.

**Conclusions:**

The work in China illustrates well the direct impact that health education can have in improving knowledge and awareness, and in changing hygiene behaviour. Further, it can provide insight into the public health outcomes of a multi-component integrated control program, where health education prevents re-infection and periodic drug treatment reduces prevalence and morbidity.

## Findings

Roundworms (*Ascaris lumbricoides*), whipworms (*Trichuris trichiura*) and hookworms (*Necator americanus* and *Ancylostoma duodenale*) are intestinal parasitic nematodes that are the scourge of many developing countries due to the fact that chronically infected individuals can succumb to a variety of clinical complications, including poor physical and mental development [1, 2]. Transmission of these soil-transmitted helminths (STHs) is associated with poverty, poor hygiene behaviour, lack of clean water and inadequate waste disposal and sanitation [3]. Consequently, high rates of infection are common in deprived urban as well as in rural areas, where people have limited or no access to health care and preventive measures [1]. Globally, an estimated 438.9 million people were infected with hookworm in 2010, 819.0 million with *A. lumbricoides* and 464.6 million with *T. trichiura* [4].

In the People’s Republic of China (PR China), the 2001–2004 second national survey of parasitic diseases indicated there were 129 million STH infections with the highest prevalence occurring in children aged 5 to 14 years, emphasising the continued importance of STHs as a significant public health problem in the country [5]. To overcome the challenge posed by STHs and other parasitic infections, the Chinese Ministry of Health announced in 2005 the “National Control Program on Important Parasitic Diseases from 2006 to 2015” which set the target of reducing parasite prevalence by 70% by the year 2015 [5].

Currently the key strategy for global STH control in endemic areas is the periodic administration of anthelminthic drugs, e.g. albendazole or mebendazole, to high risk groups, especially school-aged children [6]. While this strategy is effective in achieving morbidity control, it does not prevent re-infection, which usually occurs rapidly, and therefore is not likely to interrupt transmission as a stand-alone intervention [7–10]. Additional preventive public health measures, such as improvements in hygiene behaviour achieved through health education, are required to achieve the sustainable control of these parasitic worms.

We reported recently on the development and testing of a health education package that included a 12-minute animated narrative cartoon video entitled “The Magic Glasses” (accessible at: http://www.nejm.org/action/showMediaPlayer?doi=10.1056%2FNEJMoa1204885&aid=NEJMoa1204885_attach_1&area) to prevent STH infections in Chinese primary school students. The cluster randomized controlled trial, conducted in Linxiang City District, Hunan province, and involving 1718 children aged 9–10 years in 38 rural schools over the course of 1 school year (September 2010 through June 2011), resulted in the health education package having a 50% efficacy in preventing STH infections [11]. The trial established the proof of principle that health education can increase knowledge and change behavior, resulting in fewer intestinal worm infections. Crucial to the success of this study was the very early involvement in the study of health workers, health and education officials, teachers, parents and students, and a thorough appraisal of their knowledge, attitudes, and practices regarding STHs and the risk factors associated with infection. Consideration of all these features enabled the development of a culturally tailored, informative, and engaging educational package; but to show broader application, our findings required further validation in another epidemiological and cultural setting in China.

In 2013 we commenced a new trial of the educational package in two counties of the ethnically diverse Xishuangbanna autonomous prefecture in Yunnan province, where the force of STH infection is higher (>50% prevalence) [12] than Hunan province (10% prevalence). Xishuangbanna is located in the south of the province and borders the Lao People’s Democratic Republic and Myanmar. The prefecture has approximately 1 million inhabitants, of whom approximately 30% are Han Chinese, 30% are Dai and the remainder belong to a diverse group of other ethnic minorities. The study design is similar to that of the trial we undertook in Hunan province. Involving 9–10 year old schoolchildren, the cluster-randomised controlled trial will determine the impact of the video-based health education package on knowledge, hygiene behaviour, and STH infection outcomes. Forty schools, each with at least 38 grade 4 children, have been randomly selected in two counties, Jinghong and Menghai, both predominantly non-Han, using a spatial sampling frame to minimise contamination. Of these, 20 schools have been randomly assigned intervention status and 20 as controls. Each student in grade 4 has been recruited into the study cohort and will be followed over 2 school years. Primary endpoints will be incidence and intensity of STH infection and knowledge about the worms, their transmission, symptoms, treatment and prevention; secondary endpoints will be change in observed hand washing behaviour; tertiary endpoints will be measures of morbidity (haemoglobin levels to assess anaemia, height and weight to reflect growth and malnutrition) and educational outcomes (school academic performance and attendance). We will also evaluate the cost-effectiveness of the video-based health education package both on its own and in combination with anthelminthic chemotherapy. The costs will be compared to chemotherapy alone, helping to guide STH control policy in Yunnan, other parts of China and beyond. To date, there have been few economic evaluations of parasite control programs and, generally, these cost and cost-effectiveness analyses have evaluated treatment components only [13].

The research we are undertaking in China is designed to establish an evidence-base for a health educational package for use in schools that can be readily incorporated into the school curriculum in the framework of, for example, a regional de-worming program. The package effectively complements the current approach to STH control advocated by the World Health Organisation [6] and can, following adaptation to local cultural realities, be deployed in non-Chinese settings as well. The approach is currently being tested in Côte d’Ivoire in West Africa and we will further expand into the Philippines in 2015. Future programs could also involve the integration of chemotherapy and health education with WASH (water, sanitation and hygiene) efforts to ensure access to clean water and good sanitation in addition to improved personal hygiene [14–16]. Ultimately, the work in China has the potential to be a model for other neglected tropical diseases (NTDs) because it illustrates the direct impact that health education can have in improving knowledge and awareness, and in changing hygiene behaviour, thereby reducing the chances of an individual becoming infected. Further, it can provide insight into the public health outcomes of a multi-component integrated control program, where health education prevents re-infection and periodic drug treatment is used to reduce prevalence and morbidity.

## Conclusion

Having already successfully pioneered multi-component integrated strategies, which included health education, for the control of other NTDs such as lymphatic filariasis and schistosomiasis [17–20], the Chinese authorities are in an ideal position to undertake a multi-component integrated public health strategy that combines mass drug administration (MDA) with health education and improved WASH for the control of STH in rural China. Based on the national-scale experience, China would then be in a good position to help implement such projects in other countries.
